# Comparison of codon usage measures and their applicability in prediction of microbial gene expressivity

**DOI:** 10.1186/1471-2105-6-182

**Published:** 2005-07-19

**Authors:** Fran Supek, Kristian Vlahoviček

**Affiliations:** 1 Department of Molecular Biology, Division of Biology, Faculty of Science, Zagreb University, Rooseveltov trg 6, 10000 Zagreb, Croatia; 2 Protein Structure and Bioinformatics, International Centre for Genetic Engineering and Biotechnology, Padriciano 99, 34012 Trieste, Italy

## Abstract

**Background:**

There are a number of methods (also called: measures) currently in use that quantify codon usage in genes. These measures are often influenced by other sequence properties, such as length. This can introduce strong methodological bias into measurements; therefore we attempted to develop a method free from such dependencies. One of the common applications of codon usage analyses is to quantitatively predict gene expressivity.

**Results:**

We compared the performance of several commonly used measures and a novel method we introduce in this paper – Measure Independent of Length and Composition (MILC). Large, randomly generated sequence sets were used to test for dependence on (i) sequence length, (ii) overall amount of codon bias and (iii) codon bias discrepancy in the sequences. A derivative of the method, named MELP (MILC-based Expression Level Predictor) can be used to quantitatively predict gene expression levels from genomic data. It was compared to other similar predictors by examining their correlation with actual, experimentally obtained mRNA or protein abundances.

**Conclusion:**

We have established that MILC is a generally applicable measure, being resistant to changes in gene length and overall nucleotide composition, and introducing little noise into measurements. Other methods, however, may also be appropriate in certain applications. Our efforts to quantitatively predict gene expression levels in several prokaryotes and unicellular eukaryotes met with varying levels of success, depending on the experimental dataset and predictor used. Out of all methods, MELP and Rainer Merkl's GCB method had the most consistent behaviour. A 'reference set' containing known ribosomal protein genes appears to be a valid starting point for a codon usage-based expressivity prediction.

## Background

As the numbers of sequenced genes grew, it became evident that synonymous codons are not used equally [1-3]. Codon frequencies were found to vary on 3 levels: between genomes, between genes in the same genome, and within a single gene [4]. Many factors have been shown to influence codon usage patterns, the most important being: (i) overall nucleotide composition of the genome, reflecting mutational biases; (ii) selective forces acting on highly expressed genes to improve efficiency of translation [5]; and (iii) horizontal gene transfer, with transferred genes retaining the codon frequencies of their former host [6]. Connections have also been demonstrated between codon usage and: (i) gene length [7]; (ii) location on the chromosome [8]; (iii) the strand it resides on [9]; (iv) need for specific secondary structures in mRNA [10]; and (v) characteristics of the gene's protein product, such as its hydrophobicity [11] or secondary structure elements [12].

Moreover, the relative influence of each of these factors varies from genome to genome, and from gene to gene. For example, selection for translation efficiency shapes codon usage more in fast-growing microbes [13] than in slow-growing ones [14]. In contrast, codon usage of human genes depends largely on GC richness of the chromosomal region (isochore) [15]. It is still unclear to what extent other elements contribute to the genes' codon usage patterns [16]. The multitude of influences on codon preferences, as well as high dimensionality of codon usage data, necessitated the development of various measures (also called: statistics) of codon usage.

Many researchers in this field formulated their own measures, which led to a large number of available methods [17,18] for codon usage analysis. Unfortunately, these methods are not universally applicable, as their behaviour tends to be context-dependant. They may exhibit strong artefacts with varying (i) sequence length, (ii) overall amount of codon bias and (iii) codon bias discrepancy (see Results and Discussion for an explanation). Previous works [19,20] discussed this issue and compared some of the commonly used measures available at the time. Our aim was to develop and test a measure that would be free from dependence on the aforementioned contexts. Also, we attempted to verify the usefulness of such a measure by employing it to predict gene expressivity in microbial genomes.

## Results & discussion

### The "Measure Independent of Length and Composition" (MILC)

Our primary motivation in developing this novel method was to correct for possible artefacts due to sequence length variability. The measure should be able to quantify the distance in codon usage between a gene and some expected distribution of codons. The codon distribution could either be calculated from the background nucleotide composition, or derived from a single gene or a gene group. Therefore, MILC is conceptually similar to Karlin and Mrazek's B [21], Novembre's ENC' [19] or Urrutia and Hurst's MCB method [22].

Mathematically, the measure is based on a log-likelihood ratio score used in the statistical *G*-test for goodness-of-fit. This methodology yields numerically similar results to the more commonly used χ^2 ^test, but may hold theoretical advantages over it in statistical analyses [23]. Both of the methods have been used in past examinations of codon usage patterns [24,25].

The individual contribution *M*_*a *_of each amino acid *a *to the MILC statistic is calculated as



where *O*_*c *_denotes the actual observed count of the codon *c *in a gene, and *E*_*c *_stands for the expected count of the same codon. The *O*_*c*_/*E*_*c *_ratio is mathematically equal to, and can be replaced by *f*_*c*_/*g*_*c*_, where *f*_*c *_is the frequency of the codon c in a gene, and *g*_*c *_is the expected frequency of the same codon. The sum of *f *or *g *over all codons for each amino acid should equal 1. The total difference in codon usage is then assessed by the following formula:



The sum of contributions of all amino acids (stop codons are excluded from calculation) is divided by *L*, the gene length in codons, in attempt to compensate for the expected increase with total number of codons. This is analogous to the procedure described in [25]. However, such a „scaled χ^2^" statistic still depends on gene length [20], greatly overestimating the overall amount of bias in shorter sequences. The correction factor *C *in Equation 2 attempts to correct for this overestimation.

The cause for the abovementioned effect are sampling errors: a relatively small number of observations (counted codons) cannot exactly fit the expected distribution, leading to a higher perceived χ^2 ^score. In order to demonstrate the effect, let us presume that the expected codon frequencies for two cysteine codons are *g(UGU) *= 0.5 and *g(UGC) *= 0.5; and that our hypothetical gene complies with these codon frequencies. However, a short gene might have only a single codon for Cys, thus the observed counts can be only O_UGU _= 1 and O_UGC _= 0, or vice versa. Either way, instead of being equal to 0, the cysteine's contribution to the χ^2 ^score will be:



In case the gene has two cysteines, there is a 50% chance that O_UGU _= O_UGC _= 1, which would yield a (correct) χ^2 ^score of 0; and a 50% chance that one of them will be 2, and the other 0, which gives a χ^2 ^score of 2. The weighted average of these scores will again be equal to 1. Moving on to cases with 3, 4 or more cysteines we see that always *M*_Cys _= 1, and it can be shown that for each amino acid in this case *M*_*a *_is equal to its degree of redundancy minus 1 (e.g. *M*_Ile _= 2, *M*_Pro _= 3). In fact, this is the expected value of the χ^2 ^statistic under the null hypothesis (observed frequencies match the expected frequencies), which equals the number of degrees of freedom. The calculation can be generalized to cases when the observed frequencies do not match the expected codon distribution, and is also applicable to the *G *statistic MILC is based upon. Further examples to better illustrate this point are given in the material accompanying this paper [see Additional file 1].

To reiterate, in a situation where the gene's codon usage matches the expected distribution, with all amino acids present, the sampling errors will increase the χ^2 ^score by 41, and the „scaled χ^2^" by 41/*L*. The correction factor *C *is therefore calculated as:



where *r*_*a *_is the number of possible codons for the amino acid *a *– its degeneracy class. Only the amino acids actually present at least once in the sequence contribute to *C*, e.g. if a gene missed one of the four-fold amino acids, *C *would be 38/*L *+ 0.5. When the observed frequencies match the expected codon distribution closely, MILC can assume negative values. In order to compensate, a constant of 0.5 is added to the correction factor *C *(see Equation 4). Regarding minimum sequence length, we recommend that only sequences of 80 codons or longer be analysed using MILC (or any other measure of codon usage); many researchers set this threshold to even higher values, such as 100.

### Behaviour of codon usage measures under varying conditions

A multitude of methods to measure codon usage has been published, including "scaled χ^2^" [25], "effective number of codons" ENC [26], "codon bias index" CBI [27], "intrinsic codon bias index" ICDI [28], two versions of "codon bias" B [21,29], "maximum likelihood codon bias" MCB [22], "effective number of codons prime" ENC' [19], and "synonymous codon bias orderliness" SCUO [30]. Among those, we chose to test the methods that have been either frequently used in codon usage examinations, or that are new and haven't been extensively tested [20].

ENC is an older, widely accepted measure that quantifies the degree of deviation from equal use of synonymous codons; ENC' gives results comparable to ENC but allows comparison to any desired codon distribution; the 1998 version of Karlin and Mrazek's B has been used extensively in later research of microbial genomes by the same authors; MCB is a method conceptually similar to B, used in examinations of human genes; and SCUO is a representative of the information theory-based measures, which have recently been used on several occasions [31,32] to analyze codon usage. Finally, the method proposed in this paper, MILC, is compared in performance to the aforementioned methodologies.

**Figure 1:**
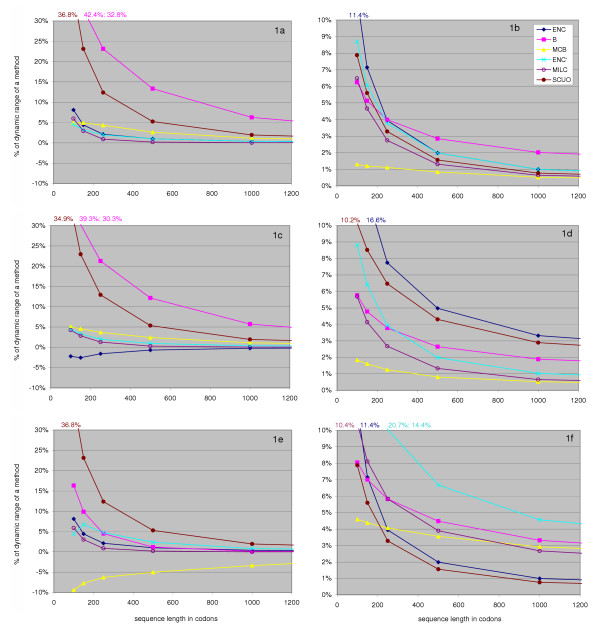
Effect of sequence length on behaviour of codon usage measures. Figure 1a, 1c and 1e illustrate the degree of misestimation of a measure at varying sequence lengths (*x *axis), compared to the values at 2500 codons.. The values were obtained by calculating means for 10000 randomly generated sequences per method per length, and are expressed as percentages of the measures' dynamic range (see Methods). Figures 1b, 1d and 1f display standard deviations of the same 10000 measurements, as percentage of the dynamic range; higher values mean a method is more 'noisy'. In Figures 1a and 1b we generated sequences unbiased in use of codons and compared them to a frequency table also assuming equal use ('None', see Methods'). In 1c and 1d both the sequences and the expected frequency were, on overall, biased ('Med-1'); in 1e and 1f the sequences were biased ('Med-1'), but were compared to an unbiased expected frequency table ('None').

Figure 1 demonstrates the behaviour of the methods when examining genes of differing lengths. Pseudorandomly generated sequences (or 'genes') obtained using INCA [33] were used for testing under varying conditions (see Methods): Figures 1a, 1c and 1e show the performance (degree of misestimation) for chosen measures at 5 different lengths, with 1b, 1d and 1f showing the standard deviations for the 10000 measurements performed at each length. In this aspect, our testing conditions resemble the ones previously used by Comeron and Aguade [20] or Novembre [19], the essential difference being the normalization and comparison of the results. Here, the values are presented as percentages of the 'dynamic range' of a measure (the largest difference between its high and low values under realistic conditions, see Methods). We feel this is more reasonable than e.g. normalizing a mean of the sample at a certain length by simply dividing it by the value at 2500 codons, which (i) unfairly penalizes measures which approach zero as bias lessens, as opposed to those approaching an arbitrary value, e.g. 61 for ENC and ENC', and (ii) among the measures approaching zero, favours those displaying larger values at 2500 codons, in spite of this being an undesirable quality – the value should be as close to zero as possible. For instance, both B and MCB are meant to equal 0 when expected and observed codon frequencies match, however in practice at the length of 2500 codons B assumes the value of approx. 0.1, and MCB of 0.033 (Table 2, "None" dataset). Dividing the misestimation of each measure by the above values would be unfairly advantageous for B; a more extreme example is ENC with its baseline value of 60.9. These issues are addressed by expressing the results as percentages of the dynamic range – a simple linear transformation essential for objective comparison of the methods' performances. However, when using a single measure to compare genes (or gene groups), or to determine association with other genomic data, it should not matter if the normalization is performed or not. The relative distances of codon usage in two genes (gene groups) would remain equal in both cases, and the degree of correlation with other genomic data would also not change.

**Table 2:** Determining the 'dynamic range' for measures of codon usage

dataset	method	max mean	coef var	dataset	method	min mean	coef var	**dyn range**
High-2	ENC	26.1757	0.3073	None	ENC	60.9141	0.1390	**-34.738**
High-2	B | None	1.0250	0.0155	None	B | None	0.0998	0.0118	**0.925**
High-2	MCB | None	3.0810	0.0783	None	MCB | None	0.0330	0.0078	**3.048**
High-2	ENC' | None	26.1757	0.3073	None	ENC' | None	60.9141	0.1390	**-34.738**
High-2	MILC | None	1.9410	0.0389	None	MILC | None	0.5000	0.0037	**1.441**
High-2	SCUO	0.5470	0.0146	None	SCUO	0.0068	0.0016	**0.540**

We designed three experiments to determine to what extent changing gene length affects each measure. In the first experiment (Figures 1a and 1b) the expected distribution assumes equal codon frequencies ("None", see Methods) and the generated sets of genes attempt to mimic that distribution. Therefore, the methods should ideally report a minimal distance between the observed and the expected distribution. ENC, ENC', MILC and MCB are generally well behaved under these conditions and tend to somewhat overestimate the amounts of bias in short sequences, MCB overestimates bias also in longer sequences. In contrast, B and SCUO greatly overestimate the bias in shorter genes (by "shorter" we assume a range of gene lengths most frequent in genomes, e.g. 100–500 codons). For example, using B on sequences 250 and 500 codons long would result in the first sequence being seemingly different twice as much from the expected distribution as the second one. Moreover, the overestimation at 250 codons may amount to as much as a quarter of the dynamic range of B. As anticipated, the variability of all measures (Figure 1b) decreases with an increase in gene length. It must be noted that MCB measurements introduce significantly less noise than the rest of the methods, particularly in short genes.

The second experiment, where the overall amount of bias in both the generated sequences and the expected distribution increases (Figure 1c) shows little change regarding length dependence – all methods see a very modest improvement in performance. ENC now tends to slightly underestimate bias, however, the variability chart (Figure 1d) shows that here it becomes noticeably less reliable than other methods, and so does SCUO. MCB is still the best performer, followed by MILC and B for shorter sequences, and ENC' for longer ones.

Figures 1e and 1f, representing the third experiment, demonstrate what happens when a gene unbiased in codon usage differs from the biased expected codon frequencies, derived from the "Med-1" dataset (see Methods). This is, in fact, a situation more likely to occur in real-life applications, as a gene would probably show at least some deviation from the expected codon distribution. ENC and SCUO expectedly behave precisely the same as in 1a and 1b, because they by definition always assume an unbiased expected distribution. Interestingly, B improves significantly and does not feel as much influence of gene length when the observed and expected codon distributions differ. It now performs on par with ENC' and MCB, both of which show a detrimental effect of increasing distance between the observed and the expected distribution. This factor also increases the amount of variation introduced by measures (excluding ENC and SCUO), most of all ENC', and causes MCB to lose its advantage over MILC and B.

**Figure 2:**
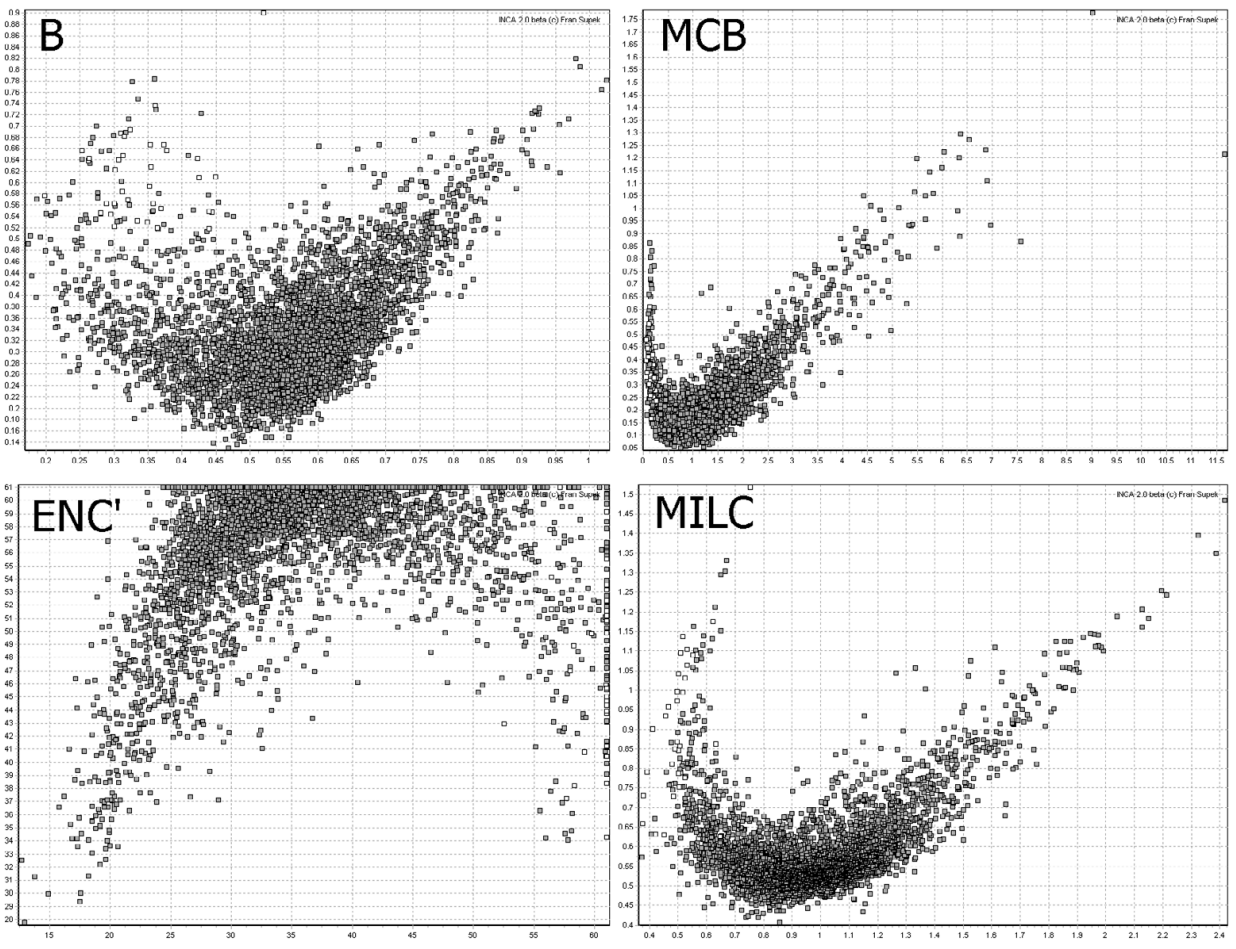
Plots of the *E. coli *genome made using different measures of codon usage. The four plots were made by using measures that allow an expected codon distribution to be specified: B, MCB, ENC' or MILC. The distance of codon usage of a gene from *E. coli *ribosomal genes was plotted on the *x *axis, and the distance of codon usage of a gene from the average codon usage of *E. coli *was plotted on the *y *axis. A characteristic 'crescent moon' shape is seen on all four plots. White square represent ribosomal protein genes, while all other genes are represented by grey squares.

We have shown that ENC and ENC' display a drop in reliability as the overall amount of bias (measured by ENC, Figure 1d), or the difference in bias (measured by ENC', 1f) increases. The explanation is the cutoff value that both measures introduce [19,26], causing the distribution of the measurements to become asymmetrical and therefore artificially reducing the measures' variance when the observed codon distribution is close to the expected one. Having such a threshold might, in theory, mask biologically relevant information; for an example, see the ENC' plot in Figure 2

Measures of codon usage introduce different levels of statistical bias in shorter genes; however, it must be noted that even if this influence were completely eliminated, there might still exist a connection between codon bias and length caused by the inherent properties of the sequences. Selection might be acting to optimize codon usage patterns (and therefore translational efficiency) in energetically costly longer genes; on the other hand it might also act to reduce the size of highly expressed (and strongly biased) proteins [7]. The only way to nullify these length effects – if this is desired – is to use regression, while employing a length-insensitive measure.

In addition to being resistant to length variation, the methods should ideally be invariant to both overall bias and the relative difference in codon usage. Moreover, the measures should be commutative with respect to properties of the observed and expected distributions. We designed two experiments to investigate these issues.

**Figure 3:**
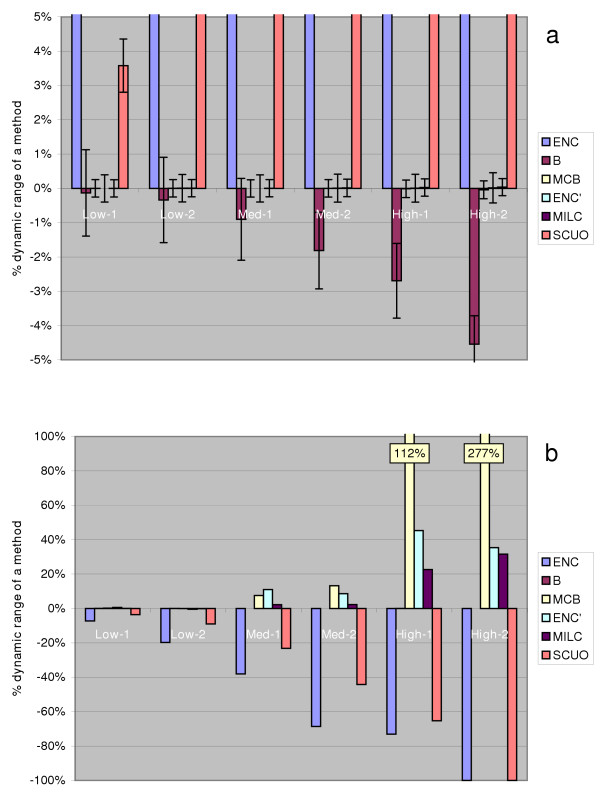
Effect of overall amount of bias on behaviour of codon usage measures. Figure 3a describes the change in behaviour of each measure as the overall bias increases from unbiased ('None') to a nucleotide composition noted on the *x *axis. The values were obtained from 10000 randomly generated sequences, 2500 codons long, per frequency table (None, Low-1, Low-2 etc.) per measure. Figure 3b demonstrates how the measures react when the nucleotide compositions of the generated sequences and the expected codon frequency table are interchanged (commutative property). See Results and Discussion for further explanation. In both figures, the values on the *y *axis are expressed as percentages of the measures' dynamic ranges.

Figure 3a shows the influence of overall amount of codon bias ('background nucleotide composition') on performance of the individual methods: we examined sets of 10000 sequences generated to match the expected frequencies at varying degrees of bias; the sequences were 2500 codons long to eliminate gene length effects. The baseline value was determined by comparing unbiased ("None") genes to unbiased ("None") expected frequencies. ENC and SCUO report higher differences from the baseline as the overall bias increases, which is anticipated since overall bias is exactly what the two methods attempt to quantify. The other methods' results should not vary between datasets. Indeed, ENC', MILC and MCB have proven to be independent of this factor, while B only slightly decreases as overall bias rises.

Furthermore, in order to test the commutative property, using each measure we compared datasets with varying levels of bias to the "None" expected distribution, and vice versa. Theoretically, when using many long sequences, comparing "None" genes to, for instance, "Med-1" expected distribution should yield the same result as comparing "Med-1" genes to the "None" expected distribution. In Figure 3b we show that among the measures that allow comparisons, the only one handling this appropriately was Karlin and Mrazek's B. MILC is less sensitive than ENC' and especially MCB, which displays a polar effect, being more strongly influenced by changes in the overall bias in the expected frequencies.

In genomes, individual amino acids may vary in amount of codon bias, an occurrence termed 'codon bias discrepancy', best described by the phrase "some codons are more optimal than others" in Fuglsang's paper [34]. For instance, in *E. coli *the CGU and CGC codons for arginine are strongly preferred over the other four codons, while six codons for serine are chosen more uniformly, with a mild preference for AGC over the others.

**Figure 4:**
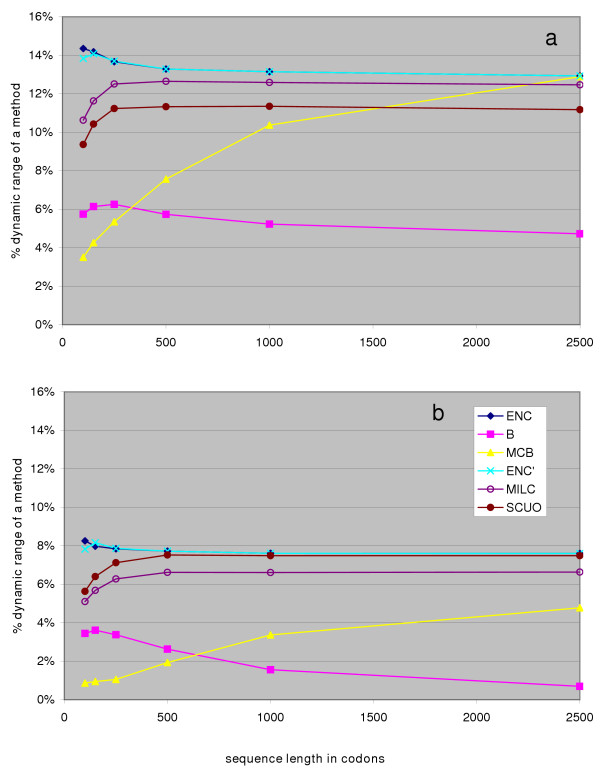
Effect of codon bias discrepancy on behaviour of codon usage measures. The figure shows how the measures react to codon discrepancy, i.e. when the amino acids within a sequence differ in amounts of bias. The value on the *y *axis is the amount of overestimation (in % of the methods' dynamic ranges) that occurs as discrepancy is introduced; this was determined by examining 10000 generated sequences for each length (*x *axis) and method. Figure 4a uses frequency tables adopted from Fuglsang [35], and 4b uses the authors' own frequency tables.

It has been implied that ENC may be dependant on the strength of the codon biasdiscrepancy [35], and the same limitations are expected to apply to the ENC' due to the similarities in calculation of the two statistics. Based on two frequency tables adopted from Fuglsang [35], representing examples of moderately biased codon distributions with and without discrepancy, we generated genes of varying lengths and compared them to a uniform distribution of codons. Figure 4a demonstrates that this amount of discrepancy causes most of the methods to moderately overestimate overall bias (10–15% of the dynamic range), while B is less affected by this change. Figure 4b illustrates a similar situation, however this time we performed the test using our own codon distribution, "Med-1d", that preserves the GC3s content of the "Med-1" while introducing discrepancy (see Methods). All of the methods again overestimated bias, although to a lesser degree; relations between methods remain similar. It is still undetermined to which extent amino acids differ in degree of bias in real genomes, and our tests do not indicate too strong an influence of this issue on measures of codon usage.

### Improving prediction of microbial gene expressivity

**Figure 5:**
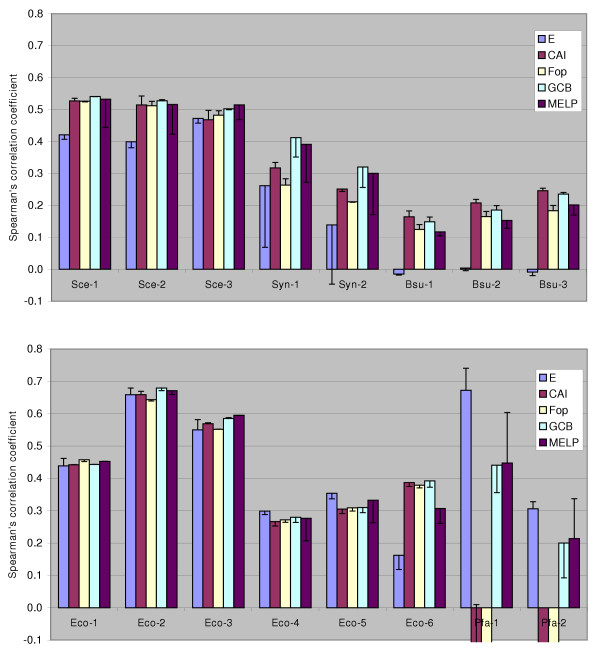
Performance of codon usage-based expression level predictors. Height of the columns shows the Spearman's (rank) correlation coefficient for each gene expression dataset / predictor combination. Error bars illustrate the change in success of the prediction when the default reference set (consisting of ribosomal protein genes >100 codons) is replaced by a computationally generated one [44].

Analogous to Karlin and Mrazek's method of predicting expression levels of genes [36], we formulate a statistic named MELP (MILC-based Expression Level Predictor), computed simply as the ratio of respective distances of a gene's codon usage from the genomic average, and a predefined reference set:



This novel method of quantitatively predicting gene expressivity is then compared to existing methods: CAI [37], F_op _[1], E [36] and GCB [38]. Instead of testing for context-independence, as we did with general measures of codon usage, we chose to rate the expression level predictors by how well they approximate real-world observations. We have collected datasets, listed in Table 3 (Methods), which consist of either mRNA or protein abundance data for unicellular organisms obtained by different methods – mostly cDNA microarrays, but also by Affymetrix arrays (Pfa-2, and partly Sce-3 data), SAGE (also partly in Sce-3), and a number of quantitative proteomics techniques. This was done in order to assemble a collection of heterogeneous data large enough to allow a rough comparison of codon usage-based predictors of gene expression. Since we wanted to avoid making any assumptions about the distributions of data in each dataset, we used a nonparametric statistic, Spearman's (rank) correlation coefficient, to quantify agreement with predicted expression levels (Figure 5). We also tried calculating Pearson (linear) correlation coefficients for the data, which in some cases showed significant improvement by log-transforming the data, however this effect was not observed consistently among datasets or expression predictors [see Additional file 1].

**Table 3:** Transcript/protein abundance data used for validation of expression level predictors

**name**	**type**	**N**	**ref**	**Web source**	**Files / accessions**	**medium**
*Saccharomyces cerevisiae*						

Sce-1	prot	2014	[51]		1.ref-abund.xls, column G	rich
Sce-2	prot	3960	[52]		nature02046-s2.xls	rich (YEPD)
Sce-3	mRNA	5432	[51]		1.ref-abund.xls, column B	combined data

*Escherichia coli *K-12 MG1655						

Eco-1	prot	138	[46]		tables A1, A2, A3	minimal
Eco-2	prot	[79]	47		columns AB, RIC	rich
Eco-3	prot	69	[47]		columns PHNppm, PSppm, NSppm	minimal (MOPS, glucose)
Eco-4	mRNA	2597	[53]		3181Table6.xls, column D	rich (LB)
Eco-5	mRNA	3685	[54]		EXPSET003: PALSP01-PALSP11	minimal (MOPS, glucose)

*Escherichia coli *K-12 W3110						

Ecj-6	mRNA	3788	[55]		ex298 – ex320, ex328-ex334	

*Bacillus subtilis*						

Bsu-1	mRNA	3581	[56]		ex745 – ex749	rich (LB)
Bsu-2	mRNA	3590	[57]		ex264, ex265, ex272, ex273, ex275, ex276, ex278 – ex286	rich (LB)
Bsu-3	mRNA	3577	[58]		ex940 – ex945	DSM

*Synechocystis sp*. PCC6803						

Syn-1	mRNA	2840	[59]		ex832 – ex839	low light conditions
Syn-2	mRNA	2840	[60]		ex22, 23, 24, 44	

*Plasmodium falciparum *3D7						

Pfa-1	prot	1068	[61]		nature01107-s1.xls	average of 4 life stages
Pfa-2	mRNA	2081	[62]		Table_1, columns I, K, Q, AB, AD, AJ, AO, AQ	average of 4 life stages

The agreement of predicted and actual protein/transcript levels varied greatly between all examined combinations of prediction method and dataset. The cause may lie in the quality of experimental data; for instance, mRNA abundances and protein 2D-PAGE data have been shown not to agree well in certain cases [39]; 2D-PAGE as a method may only be suitable for detection of abundant proteins [40], while microarray data tends to suffer from noise introduced at each step of different experimental protocols [41]. The other probable reason for relatively incoherent results is that a model for predicting gene expression from genomic data, based solely on codon usage, is oversimplified. Other factors, such as promoter strength and gene copy number should also be taken into account. Fortunately, optimal codon usage in genes seems to coincide with factors enhancing transcription – this is why it is possible to observe a correlation between codon usage (acting at translation level) and transcript abundances. Keeping these limitations in mind, it seems safe to say that, in comparison to other predictors, GCB and MELP behave more consistently throughout all datasets.

Transcript and/or protein levels in a cell are normally subject to regulation, as opposed to codon usage patterns, which are 'hard-coded' in the genome sequence. If we suppose the major force shaping gene-specific codon usage patterns in microbes is selection for translation efficiency, which operates in periods of fast competitive growth, it follows that codon usage will be 'optimised' for genes highly expressed in such periods. For that reason we chose datasets of organisms harvested in exponential growth phase, and without severe nutritional restrictions in the medium. For instance, the Bsu-2 datasets describes *Bacillus *harvested at OD_600 _≅ 0.4 – 0.6; an analogous dataset [see Additional file 1] for bacteria harvested at OD_600 _≅ 1.1 does not correlate so well with predicted expression levels (Pearson's correlation coefficient for MELP = 0.234 vs. 0.187, for GCB = 0.277 vs. 0.185). In addition, the growth conditions should match the organism's natural habitat. For instance, *E. coli *grown in a rich medium has gene expression levels closer to the predicted values than *E. coli *in a defined medium; should the data in Eco-2 dataset be replaced with data from MOPS+glucose grown cells [see Additional file 1], the Pearson's correlation coefficient for log-transformed data drops from 0.720 to 0.663 (MELP), or from 0.708 to 0.642 (GCB). Furthermore, nitrogen or phosphorus starvation of *E. coli *in the Eco-3 dataset reduces the correlation with predicted values (data not shown). Such connections between codon usage and gene expression under different conditions can be used to hypothesize about the exact 'natural' environment of a microbe [42].

Any codon usage-based prediction of gene expression relies on a prior definition of a 'reference set', consisting of highly expressed genes. Our reference sets were defined as all genes coding for ribosomal proteins, longer than 100 codons; other approaches to this issue exist. For instance, the original definition for CAI [37] listed a set of genes which have been empirically proven to be highly expressed in yeast and *E. coli*; Karlin and Mrazek [36] included transcription/translation related factors and chaperones in the reference set, in addition to the ribosomal protein genes; attempts have been made to detect major trends in codon usage by iterative computational methods [38,43] and use the results to define a reference set. We investigated to what extent reference set composition affects prediction of gene expression; the alternative reference sets used were obtained from Merkl [44] and generated by computationally detecting the major trend in codon usage in a genome. The sets normally contained ribosomal protein genes, elongation factors and energy metabolism genes; also photosynthesis genes in *Synechocystis *and histones in *P. falciparum*; such functional assignments for reference set genes were not unexpected. Under the assumption that the major trend is due to translational selection, the change in reference set composition should have theoretically resulted in improved prediction. However, the outcome was highly dependent on the genome examined, and the predictor used (shown as error bars in Figure 5). In some instances, the use of the alternative reference set resulted in poorer correlation. More high-quality transcript/protein abundance data would be required to reach a definite recommendation on forming a reference set.

## Conclusion

We introduce a novel method, based on a corrected log-ratio chi-squared statistic, of measuring codon usage bias in genes or gene groups – MILC. By comparing its performance to other commonly used measures of codon usage in a variety of contexts, we have established that MILC is a generally applicable method, being resistant to changes in gene length and overall nucleotide composition, and introducing little noise into measurements. Other measures, however, may also be appropriate for specific purposes: B, when comparing very long sequences (groups of genes, whole genomes) which are expected to differ significantly in codon usage and/or exhibit bias discrepancy; or MCB, when comparing sequences of varying lengths but relatively similar in codon preferences. We have also evaluated the methods' ability to estimate gene expression levels by comparing them to actual mRNA/protein abundance data from several species. Out of the tested predictors, GCB and MELP exhibit the most consistent behaviour. A reference set defined simply by including ribosomal protein genes appears to be a valid starting point for expression level predictions in examined prokaryotes and unicellular eukaryotes, although one should be cautious when interpreting the results of such estimations. The MILC and MELP methods have been implemented in the version 2 of the INCA software, available from the bioinfo-hr.org website [45].

## Methods

### Performance evaluation

The measures of codon usage ENC, B, MCB, ENC' and SCUO were computed as in [26,21,22,19] and [30] respectively. The test sets of randomly generated sequences follow the nucleotide compositions proposed in [20], and are reviewed in Table 1. The amino acid frequencies were kept proportionate to their degeneracy class (number of codons coding for it in the standard genetic code), i.e. a 4-fold amino acid is used twice as often as a 2-fold amino acid. As a consequence of the imposed restriction on amino acid composition, the nucleotide ratios in Table 1 reflect the nucleotide composition at silent sites only. For each combination of gene length (100, 150, 250, 500, 1000 and 2500 codons) and nucleotide composition used, 10000 sequences were generated; each sequence was compared, using all measures, to an expected frequency table (derived from data in Table 1) and the mean and standard deviation for all measurements were determined. Generated sequences did not contain stop codons.

**Table 1:** Nucleotide composition of the generated sequences at silent sites

	None	Low-1	Low-2	Med-1	Med-2	High-1	High-2
f(A)	0.250	0.200	0.200	0.125	0.125	0.050	0.050
f(G)	0.250	0.300	0.200	0.375	0.125	0.450	0.050
f(C)	0.250	0.300	0.400	0.375	0.125	0.450	0.850
f(T)	0.250	0.200	0.200	0.125	0.125	0.050	0.050

Values in Figures 1, 3 and 4 are expressed as percentages of the 'dynamic range' of a method, the largest difference between its high and low values under realistic conditions. This was assessed by comparing, using each method, first a set of 10000 'None' sequences (2500 codons long) to the 'None' frequency table, and then a set of 10000 'High-2' sequences (2500 codons long) to the 'None' frequencies, and finally by subtracting the numbers; this process is summarized in Table 2. Because of this normalization process, positive values of the mean always signify overestimation of bias, even though, for instance, a higher value of ENC' normally means less bias.

The codon frequency tables used to generate sequences, derived from the None, Low, Med and High nucleotide compositions, are available in the accompanying materials [see Additional file 1], as well as the frequency tables used to test for codon usage discrepancy effects.

### Predictors of gene expression

The expression level predictors CAI, E, and GCB were computed as in [37,36] and [38], respectively. When calculating the 'frequency of optimal codons' F_op_, a codon with a relative adaptiveness (codon frequency divided by the frequency of the most frequent codon) larger than 0.9 was considered optimal. Experimental datasets used to investigate the performance of the predictors are listed in Table 1. Datasets **Sce-1, 2, 3**, and **Eco-4 **were used 'as-is' from the respective sources. **Eco-1 **dataset was created by combining molar abundances (column "N-abd") from Tables a1, a2 and a3 in [46]; if a gene occurred in more than one table, its final abundance value was calculated as an average of the two/three measurements. **Eco-2 **dataset was created from the *E. coli *Gene-Protein Database [47] by multiplying values in the "AB" column (abundances) with values in the "RIC" column (rich media) and dividing by the "MWc" column to obtain molar abundances. **Eco-3 **dataset was created by averaging the "PHNppm", "PSppm" and "NSppm" (control groups for phosphorus and nitrogen starvation experiments), and by dividing by the "MWc" column. **Ecj-6**, **Bsu-1, 2, 3**, **Syn-1 **and **Syn-2 **datasets were downloaded from the KEGG expression data repository [48] and were processed in the following manner: the local background ("Control-bkg") was subtracted from the signal intensity ("Control-sig") for each microarray spot in the control groups, and the resulting values were normalised to the sum of 10^6 ^per experiment. Finally, for each spot/gene a median value over all experiments in a dataset was calculated. The **Pfa-1 **dataset was created by averaging the sequence coverage of a protein over all four life stages; if a protein was not detected in a *P. falciparum *stage, its sequence coverage was assumed to equal 0. To create the **Pfa-2 **dataset, the columns I, K, AB and AD were averaged to obtain an mRNA abundance for the trophozoite, Q and AJ for the merozoite; column AO provided values for the gametocyte, and column AQ for the sporozoite. The final abundance values were again obtained by averaging the four life stages. Files containing coding regions of genes were downloaded from the NCBI ftp site [49] for the Eco, Sce, Pfa and Syn datasets, and from the KEGG ftp site [50] for the Ecj and Bsu datasets.

## Authors' contributions

FS devised, tested and implemented the MILC and MELP methods. KV supervised the project and contributed in biological expertise. Both authors read and approved the final manuscript.

## Supplementary Material

Additional File 1 Rationale behind the length correction of the MILC method, codon frequencies used for testing of codon usage measures, and performance of the expression level predictors. Sheets 1a, 1b and 1c demonstrate, by example, how the chi-square and G scores for amino acids of different degeneracy classes behave when the observed codon counts are small. Sheet 2 contains the codon frequency tables used in testing of the codon usage measures. Sheet 3 describes the performance of the expression level predictors, expressed as Spearman (rank) and Pearson (linear) correlation coefficients of the predicted values and experimentally obtained mRNA/protein abundance data sets. Click here for file
