# Correction: Comparison of codon usage measures and their applicability in prediction of microbial gene expressivity

**DOI:** 10.1186/1471-2105-11-463

**Published:** 2010-09-16

**Authors:** Fran Supek, Kristian Vlahoviček

**Affiliations:** 1 Division of Electronics, Rudjer Boskovic Institute, Zagreb, Croatia; 2 Division of Biology, Faculty of Science, University of Zagreb, Zagreb, Croatia; 3 Department of Informatics, University of Oslo, Oslo, Norway

## Correction

In the original manuscript [1], Equations (1) and (4) were erroneous and are given below in their correct form:

(1) $$M_{a}=2\sum_{c}O_{c}\ln\frac{O_{c}}{E_{c}}=2\sum_{c}O_{c}\ln\frac{f_{c}}{g_{c}}$$

The text referring to equation (1) states the value should be computed as the G-test statistic, which equals to 2*sum(O*ln(O/E)); therefore, the accompanying text is correct.

(4) $$C=\frac{\sum_{a}\left(r_{a}-1\right)}{L}-0.5$$

The text "a constant of 0.5 is *added to *the correction factor C" in the paragraph following equation (4), should state: "a constant of 0.5 is *subtracted from *the correction factor C".

The error in the formulae does not affect the results in the paper regarding performance of MILC, MELP and other codon distance measures as all calculations were performed using a correct implementation of MILC in the INCA software [2].
